# The spectrum of Apert syndrome: phenotype, particularities in orthodontic treatment, and characteristics of orthognathic surgery

**DOI:** 10.1186/1746-160X-3-10

**Published:** 2007-02-08

**Authors:** Ariane Hohoff, Ulrich Joos, Ulrich Meyer, Ulrike Ehmer, Thomas Stamm

**Affiliations:** 1 Department of Orthodontics, University Hospital, Westfalian Wilhelms-University, Münster, Germany; 2 Department of Craniomaxillofacial Surgery, University Hospital, Westfalian Wilhelms-University, Münster, Germany; 3 Department of Cranio-and-Maxillo Facial Surgery, Heinrich Heine University, Düsseldorf, Germany

## Abstract

In the PubMed accessible literature, information on the characteristics of interdisciplinary orthodontic and surgical treatment of patients with Apert syndrome is rare. The aim of the present article is threefold: (1) to show the spectrum of the phenotype, in order (2) to elucidate the scope of hindrances to orthodontic treatment, and (3) to demonstrate the problems of surgery and interdisciplinary approach.

Children and adolescents who were born in 1985 or later, who were diagnosed with Apert syndrome, and who sought consultation or treatment at the Departments of Orthodontics or Craniomaxillofacial Surgery at the Dental School of the University Hospital of Münster (n = 22; 9 male, 13 female) were screened. Exemplarily, three of these patients (2 male, 1 female), seeking interdisciplinary (both orthodontic and surgical treatment) are presented. Orthodontic treatment before surgery was performed by one experienced orthodontist (AH), and orthognathic surgery was performed by one experienced surgeon (UJ), who diagnosed the syndrome according to the criteria listed in OMIM™.

In the sagittal plane, the patients suffered from a mild to a very severe Angle Class III malocclusion, which was sometimes compensated by the inclination of the lower incisors; in the vertical dimension from an open bite; and transversally from a single tooth in crossbite to a circular crossbite. All patients showed dentitio tarda, some impaction, partial eruption, idopathic root resorption, transposition or other aberrations in the position of the tooth germs, and severe crowding, with sometimes parallel molar tooth buds in each quarter of the upper jaw.

Because of the severity of malocclusion, orthodontic treatment needed to be performed with fixed appliances, and mainly with superelastic wires. The therapy was hampered with respect to positioning of bands and brackets because of incomplete tooth eruption, dense gingiva, and mucopolysaccharide ridges. Some teeth did not move, or moved insufficiently (especially with respect to rotations and torque) irrespective of surgical procedures or orthodontic mechanics and materials applied, and without prognostic factors indicating these problems. Establishing occlusal contact of all teeth was difficult. Tooth movement was generally retarded, increasing the duration of orthodontic treatment. Planning of extractions was different from that of patients without this syndrome.

In one patient, the sole surgical procedure after orthodontic treatment with fixed appliances in the maxilla and mandible was a genioplasty. Most patients needed two- jaw surgery (bilateral sagittal split osteotomy [BSSO] with mandibular setback and distraction in the maxilla). During the period of distraction, the orthodontist guided the maxilla into final position by means of bite planes and intermaxillary elastics.

To our knowledge, this is the first article in the PubMed accessible literature describing the *problems *with respect to interdisciplinary orthodontic and surgical procedures. Although the treatment results are not perfect, patients undergoing these procedures benefit esthetically to a high degree.

Patients need to be informed with respect to the different kinds of extractions that need to be performed, the increased treatment time, and the results, which may be reached using realistic expectations.

## Background

Apert syndrome, which was well described by a number of authors earlier [1,2] is a rare congenital anomaly, appearing with a frequency of 1 in 55,000 to 90,909 live births [1,3,4] is one of the five craniosynostosis syndromes caused by allelic mutations of the fibroblast growth-factor receptor 2 (FGFR2) [5].

Externally, this progessive disease of growth is characterized by acrocephaly, syndactyly, and typical features in the orofacial region [6].

The lips are crossbow-shaped or trapezoidal, and the lower lip may seem to protrude [7].

The midface is retruded, which is already marked at birth [8]. The Class III discrepancy is progressive [9], the maxilla is hypoplastic [10] in all three dimensions, [7] and anteriorly there is edge-to-edge contact or an open bite [8,11,12].

Cephalometrically, a common craniofacial asymmetry can be diagnosed [13]. Maxillary height is extremely short, and upper facial height markedly reduced, whereas total facial height is increased as compared with the norm [11].

The teeth are crowded [12], and there is a mean dental developmental delay of 0.96 years, with a range of 0.5 to 2.9 years [14]. The delayed eruption may be secondary to the crowding, stacking, and displacement of the teeth within the alveolus, which is often wide to accommodate the tooth buds in two rows [7]. A primary defect in tooth eruption secondary to the "mesenchymal disorder" in Apert syndrome also must be considered [7].

In the mandible, thickening of the gingiva and delayed eruption are also observed, but to a lesser degree [7].

There are bulbous lateral swellings in the palate, which until now were not properly named in the anatomic nomenclature [15] and which might be so prominent that they become a food trap [7]. This posterior notching [7] could suggest a cleft palate [12], but is actually a *pseudo cleft palate*. A true cleft palate occurs in 23.5% of the cases [4]. A cleft soft palate or bifid uvula may be present in approximately 75% of the patients [16].

The morphological defects may induce functional defects, such as sleep apnea, disturbances of breathing, feeding, speech [17], and lip closure [7].

Despite the intrasyndromic similarities, there is a high degree of variable phenotypical expression [4,10,18].

The aim of the present case study is to show

- the clinical variablity of Apert syndrome (spectrum of the phenotype),

- implications for hindrances in orthodontic therapy, and

- characteristics of surgery and interdisciplinary treatment approach.

Table 1 gives an overview of the general treatment concept for patients with Apert syndrome at the University Hospital of Münster, which allows for individual adaptions.

**Table 1:** Interdisciplinary treatment concept for patients with Apert syndrome at the University Hospital of Münster (individual adaptions allowed).

Stage	Age	Procedure
1	3–6 months	Eradicative-osteoclastic method [35]

2	7–10 years	LeFort III osteotomy + Frontal advancement + Protraction face mask immediately postoperatively for 6–12 months (rapid stenosis, problems with anchorage of the orthodontic appliance due to exfoliation of deciduous teeth, and eruption of permanent teeth) Early LeFort III osteotomy might help to avoid a (second) LeFort III osteotomy at stage 5, and thus reduce surgical risks.

3	> 9 years	Extraction therapy

4	> 10 years	Orthodontic treatment (preparation for orthognathic surgery), retention

5	> 16 years	Orthognathic surgery

This paper refers to treatment stages 3 to 5 given in Table 1.

## Methods

Children and adolescents who were born in 1985 or later, who were diagnosed with Apert syndrome, and who sought consultation or treatment at the Department of Orthodontics or Craniomaxillofacial Surgery at the Dental School of the University Hospital of Münster (n = 22; 9 male, 13 female) were screened.

Exemplarily, three of these patients (2 male, 1 female), seeking interdisciplinary (both orthodontic and surgical treatment) were chosen because their malocclusions were representative of the whole clinical spectrum of Apert syndrome and their treatment at stages 3–5 (Table 1) thus covered the whole therapeutic spectrum, ranging from orthodontic treatment and genioplasty only to two-jaw surgery with distraction (Table 2). Orthodontic treatment of these patients was performed by one experienced orthodontist (AH); orthognathic surgery was executed by one experienced surgeon (UJ), who diagnosed the syndrome according to the criteria listed in Online Mendelian Inheritance in Man [19].

**Table 2:** Essentials of treatment of 3 patients in this study.*

**Case**	**Sex**	**Age of insertion of fixed orthodontic appliance (years)**	**Treatment time with fixed appliance (years)**	**Age at surgical intervention (years)**	**Kind of surgery**
1	Female	11.4	3.0	13.3	Genioplasty
2	Male	13.7	1.4	Planned, but not yet performed	Planned, but not yet performed: maxilla: distraction; mandible: BSSO†/mandibular setback
3	Male	15.2	1.3	18.6	Maxilla: distraction (advancement + rotation); mandible: BSSO†/mandibular setback

*These patients were chosen exemplarily because their malocclusions were representative of the whole clinical spectrum of Apert syndrome and their treatment at stages 3–5 (see Table 1) thus covered the whole therapeutic spectrum ranging from orthodontic treatment and genioplasty exclusively to 2-jaw surgery with distraction.

† BSSO = bilateral sagittal split osteotomy.

## Case descriptions

### Case 1

History (treatment stages 1 and 2 according to Table 1):

- < Age 3.0: (Surgical) treatment alieno loco.

- Age 6.2: LeFort III surgery and protraction mask.

The treatment sequence is illustrated in figures 1, 2, 3, 4, 5, 6, 7, 8.

**Figure 1:**
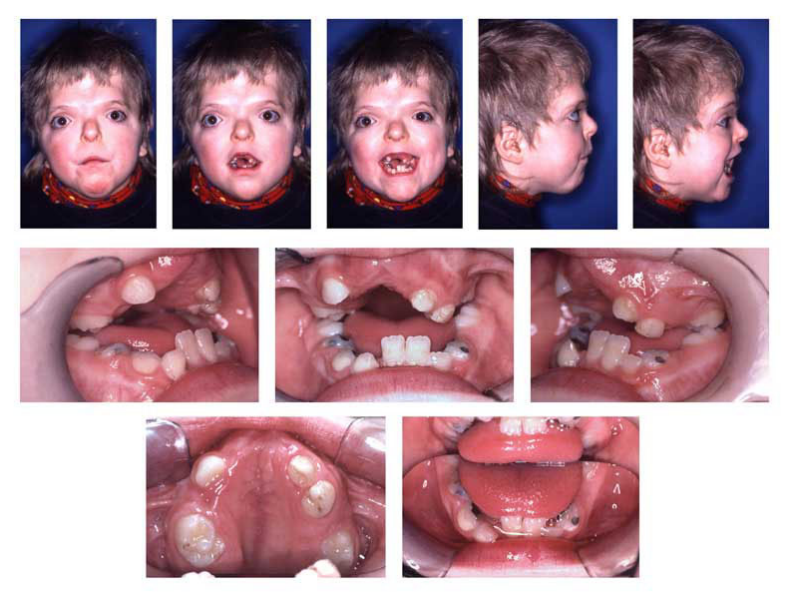
Age 8.8. Extraorally: Hypertelorism, vertical excess of the lower third of the face, trapezoidal upper lip, forced lip closure possible, but difficult. Intraorally: Dentitio tarda, crowding, Angle Class III with dental compensation by retrusion of lower incisors, circular open bite with unilateral crossbite.

**Figure 2:**
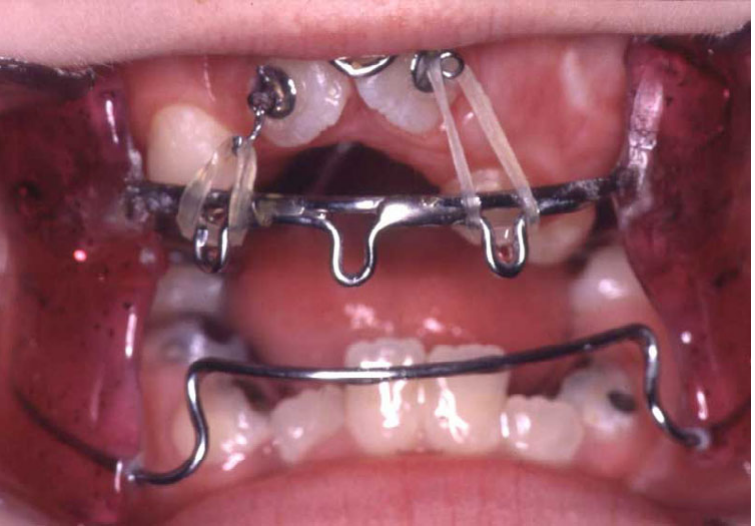
Age 9.4. Situation after surgical exposure of upper central incisors at age 9.1. Extrusion of upper central incisors with a modified functional appliance (Fränkel 3), which was not applied to treat the Class III, but only to extrude the upper central incisors and to stretch the soft tissues to facilitate lip closure.

**Figure 3:**
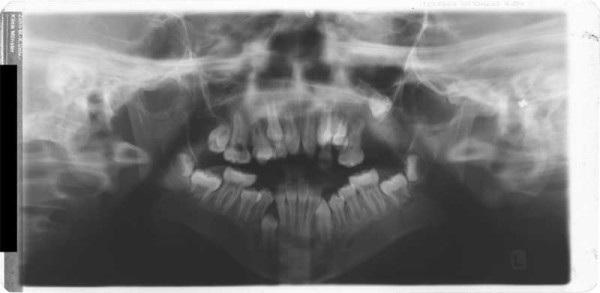
Age 10.8. Panoramic x-ray. Situation after extrusion of upper central incisors. Lack of space for 13, 23, 38, 33, 43, and 48; transposition of 22 and 23, second (supernumerary) canine in region 24, agenesis of 25. Ectopic position of 27.

**Figure 4:**
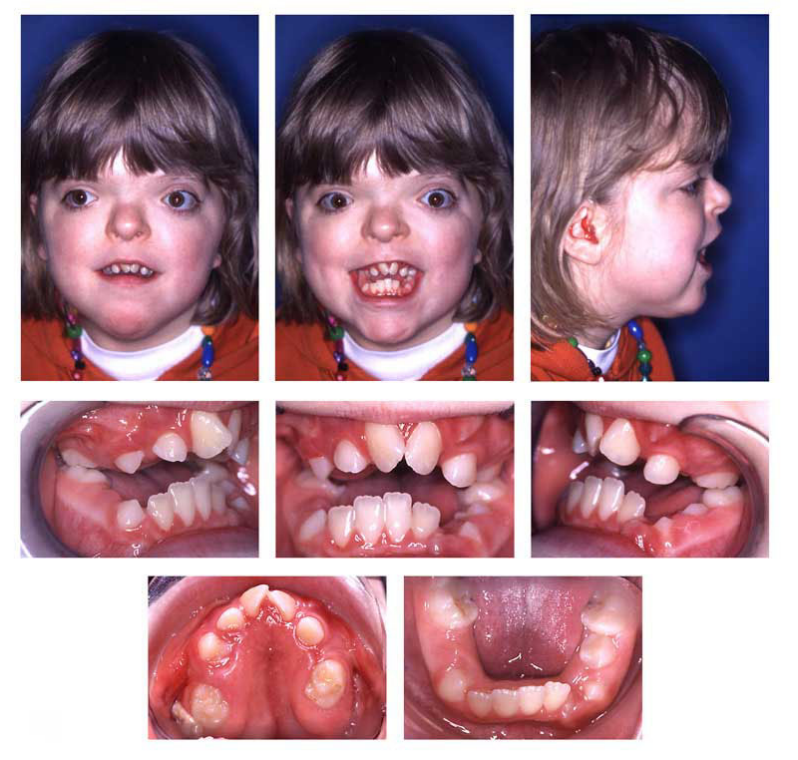
Age 10.8 Situation after surgical exposure of 11 and 21, and orthodontic extrusion of these teeth with a removable appliance from age 9.1 to 10.3 (compare Fig. 2).

**Figure 5:**
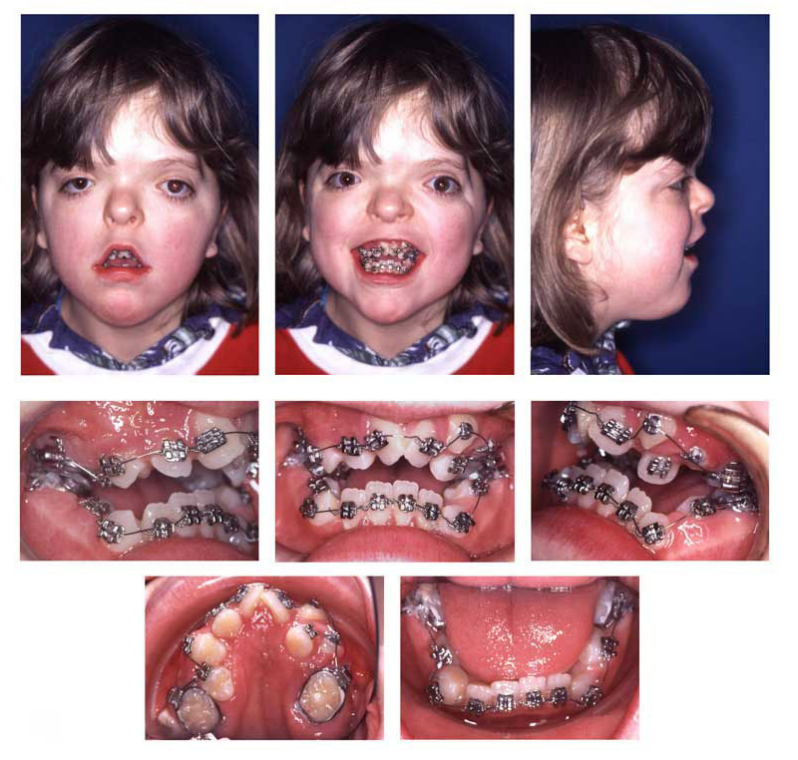
Age 11.4. Day 0 in braces. Situation after surgical exposure of 15, 24, and 45, and extraction of 13, (supernumerary) 23, 33, and 43. Syndrome-related extraction decisions, different from that in "traditional" extraction cases, were made: because tooth movement could not be predicted, it could have happened that after extraction of premolars (which had already proceeded further to the occlusal plane; compare Fig. 3), the canines, which have a lower eruption grade, could not have been moved orthodontically to the occlusal plane (compare Fig. 3). Finally, this would have resulted in lack of two teeth (canine and first premolar) in each quadrant. Therefore, because of the risk of immobility the canines were extracted instead of the first premolars, despite their guiding function and long roots, i.e. despite their high orthodontic, periodontal and prosthetic value. Transposition of 22 and 23 was accepted to remain untreated. Due to tooth position or eruption grade, placement of bands, and brackets 15, 35, and 45, was difficult. Enormous vertical distances between the upper brackets required that the archwire was not ligated to all attachments initially.

**Figure 6:**
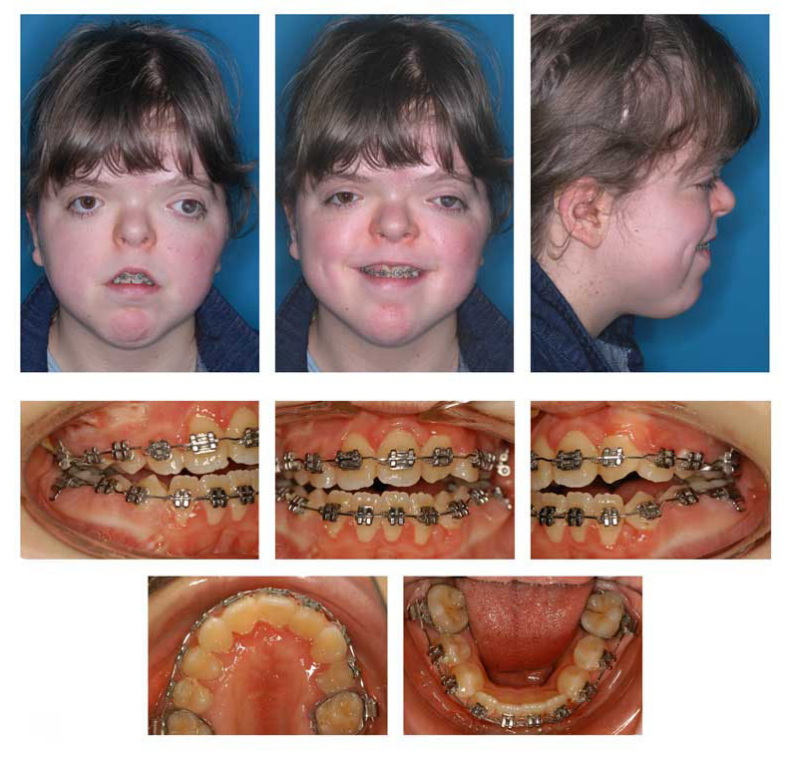
Age 13.2. After 21 months in braces. Open-bite reduction was obtained by second order bends and vertical elastics. Please note transposition of 22 and 23. Upper teeth follow the lower lip smile line. Two months before genioplasty.

**Figure 7:**
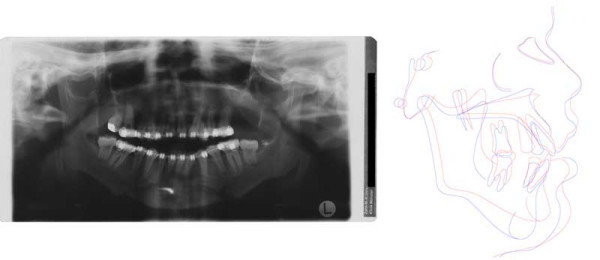
Age 13.3. After 22 months in braces and genioplasty. Left: Panoramic radiograph does not reveal root resorption. Teeth 27, 38, and 48 were removed at time of genioplasty. Right: Superimposition of lateral ceph before treatment with the fixed appliance (blue) (age 11.4) and after genioplasty (red) (age 13.3).

**Figure 8:**
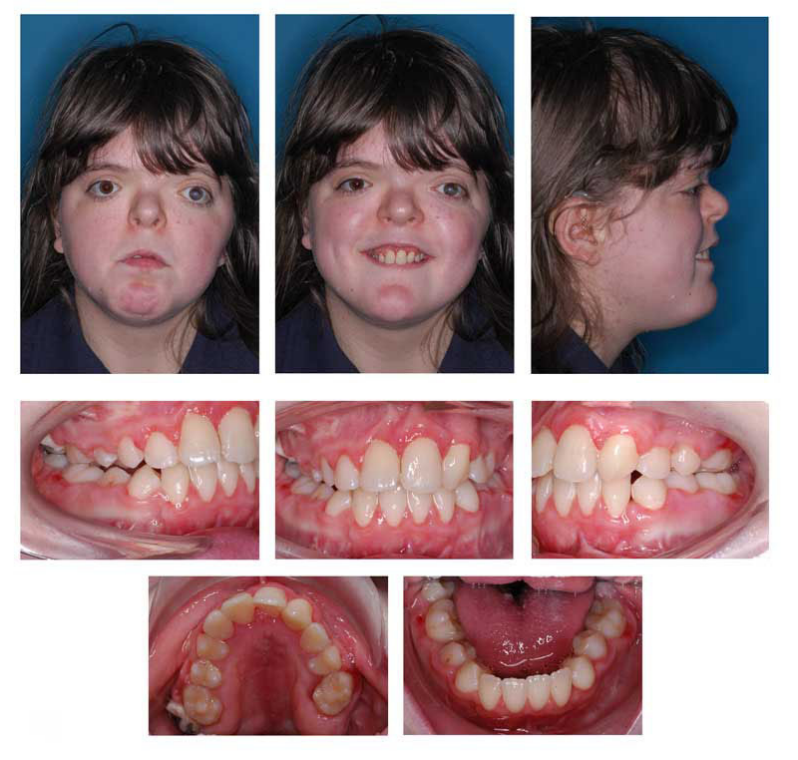
Age 14.4. End of treatment after 36 months. The upper and lower dental midline are not congruent. Torque of teeth 21 and 23 and occlusal contact between right premolars could not be established. Patient declined contouring/composite reshaping of teeth 22 and 23. Complications during treatment were maintaining oral hygiene and loss of 4 braces.

### Case 2

History (treatment stages 1 and 2 according to Table 1):

- < Age 7.0: Surgical treatment alieno loco.

- Age 7.2: Frontal advancement and protraction mask coincident with orthodontic extrusion of 11 and 21.

**Figure 9:**
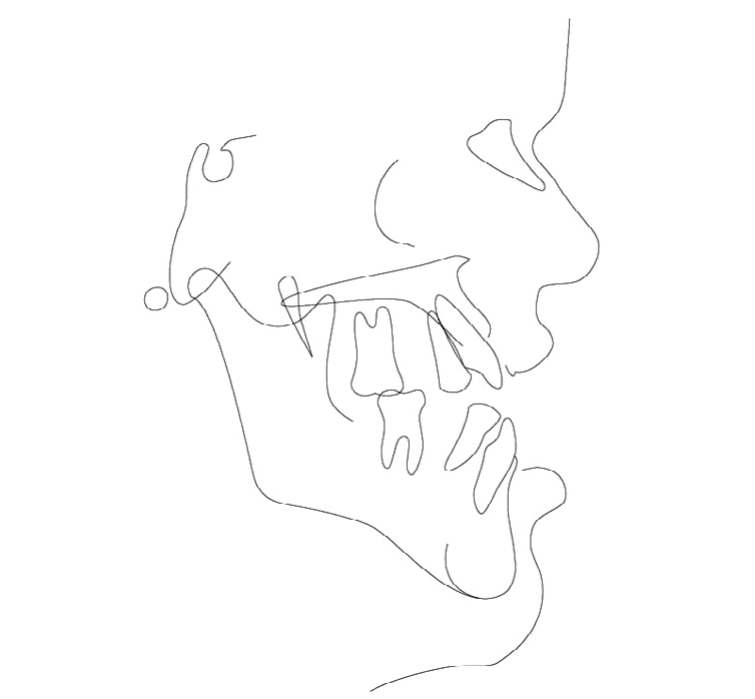
Age 10.7. Tracing of lateral x-ray. Class III, vertical excess of the lower third of the face, anterior open bite.

**Figure 10:**
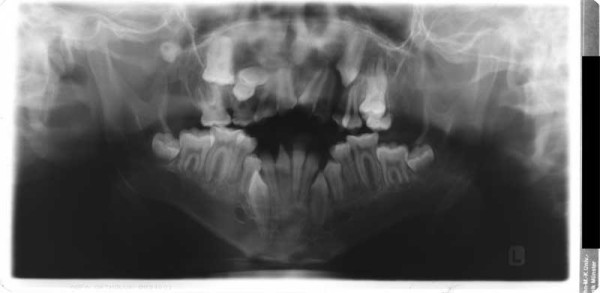
Age 13.2. Panoramic x-ray. Congenitally missing tooth 28 and impacted teeth 18, 17, 14, 13, 23, 27, 38, 33, 43 and 48. Because of extreme crowding, impaction, and space discrepancy, extraction of teeth 17, 14, 13, 23, 38, 33, 43, and 48 were considered. Tooth 18 will be left in place because of its malposition.

**Figure 11:**
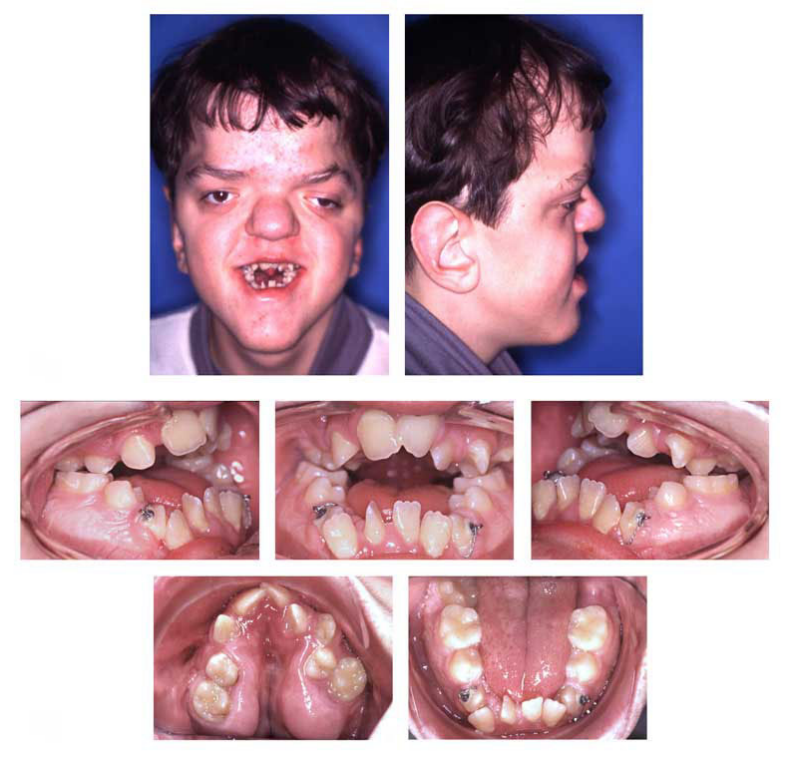
Age 13.7. Situation after surgical exposure and orthodontic extrusion of 34 and 44 with a removable appliance and before bonding of the fixed appliance. Extraorally: Hypertelorism and proptosis. The latter could be seen as a consequence of midfacial hypoplasia and retroposition of infraorbital rim. Trapezoidal shape of mouth, impossibility of lip closure at rest due to vertical excess of lower third of face. Intraorally: Class III with reclined lower incisors, anterior open bite, bilateral crossbite, and crowding in both arches. V-shaped maxillary arch with midline "pseudocleft" due to severe fibrous hyperplasia of lateral palatine ridges.

**Figure 12:**
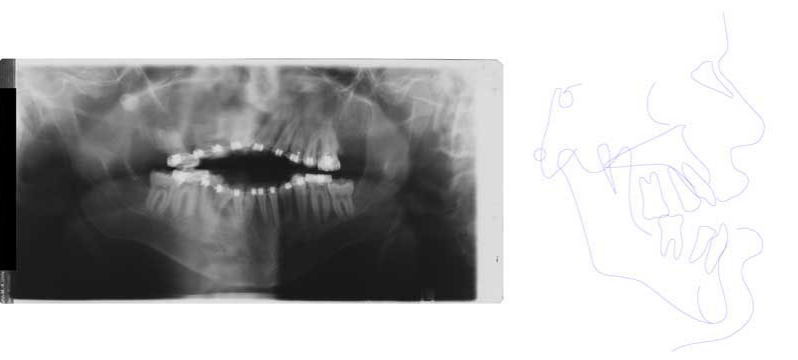
Age 14.2. After five months in braces. Panographic x-ray and tracing of lateral x-ray. Because of mandibular growth and orthodontic decompensation (the latter is a prerequisite for orthognathic surgery) and insufficient growth of maxillary complex, the open bite and Class III worsened as compared with lateral x-ray at age 10.7 (compare Fig. 10). Tooth 18 will be left in place because of its malposition.

**Figure 13:**
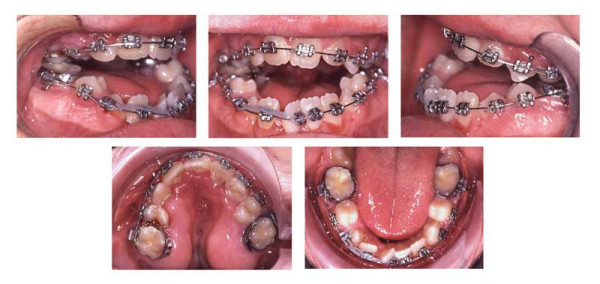
Age 14.3. After seven months of treatment. Note the mechanics for mutual derotation of teeth 41 and 44.

**Figure 14:**
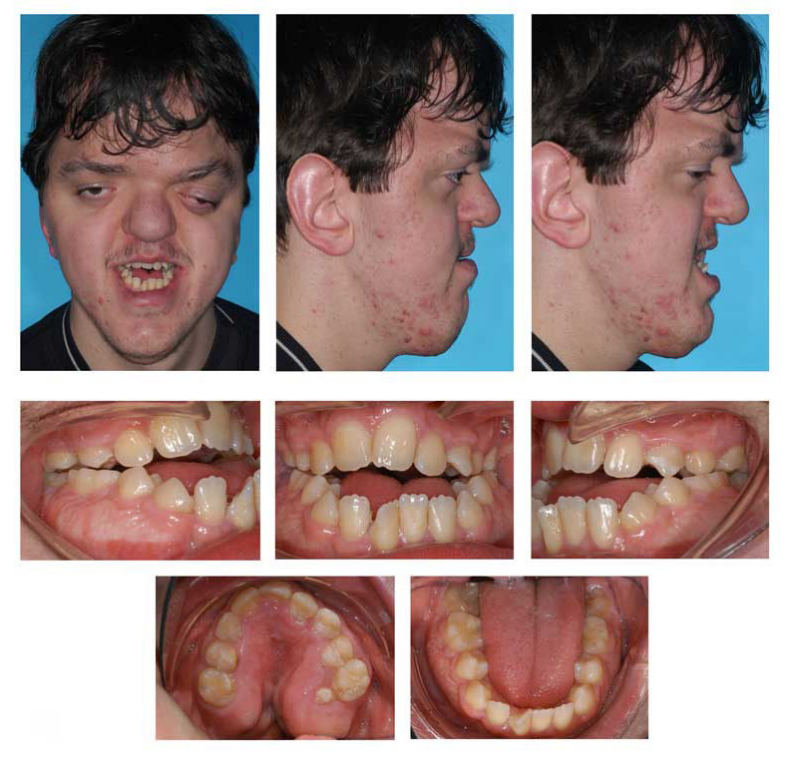
Age 16.1. End of treatment after 29 months. Tooth positions in both jaws are coordinated so that later surgical positioning into an acceptable occlusion will be possible. Teeth 27, 37, and 47 will be without antagonistic contact after orthognathic surgery and will be extracted at least 6 months before operation. Because of further growth and the requisite decompensation for orthognathic surgery, the initial Class III worsened. Complications during orthodontic treatment were a generally decelerated tooth movement, the impossibilty to establish torque in teeth 21, 22, and 24, and the impossibilty to rotate tooth 41 despite supracrestal fibrotomy, application of rotational pads, first order bands, power chains with lasso technique, and superelastic wires. Further problems during orthodontic treatment: Maintaining oral hygiene; loss of 1 bracket and loosening of 1 band; patient did not wear his retainers properly, resulting in the need for tooth grinding before surgery to enable correct positioning after elongation of some teeth.

The treatment sequence is illustrated in figures 9, 10, 11, 12, 13, 14.

**Figure 15:**
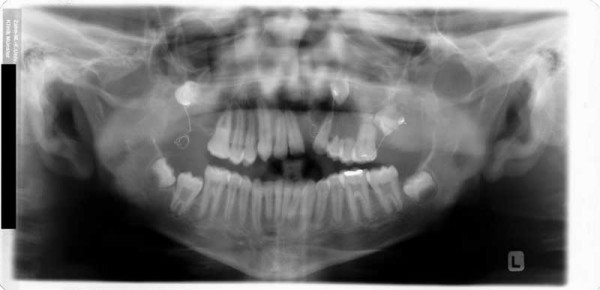
Age 14.3. Panographic x-ray. Situation after extraction of 21 due to internal resorption at age 13.5. Situation after surgical removal of 18 and 28 and surgical exposure and bonding of 17, 23, and 27; before attempting orthodontic extrusion of 17, 23, and 27. Note severe idiopathic resorption of teeth 24 and 25.

### Case 3

History (treatment stages 1 and 2 according to Table 1):

- < Age 8.5: (Surgical) treatment alieno loco

- Age 8.5: LeFort II and face mask.

The treatment sequence is illustrated in figures  15, 16, 17, 18, 19, 20, 21, 22, 23.

**Figure 16:**
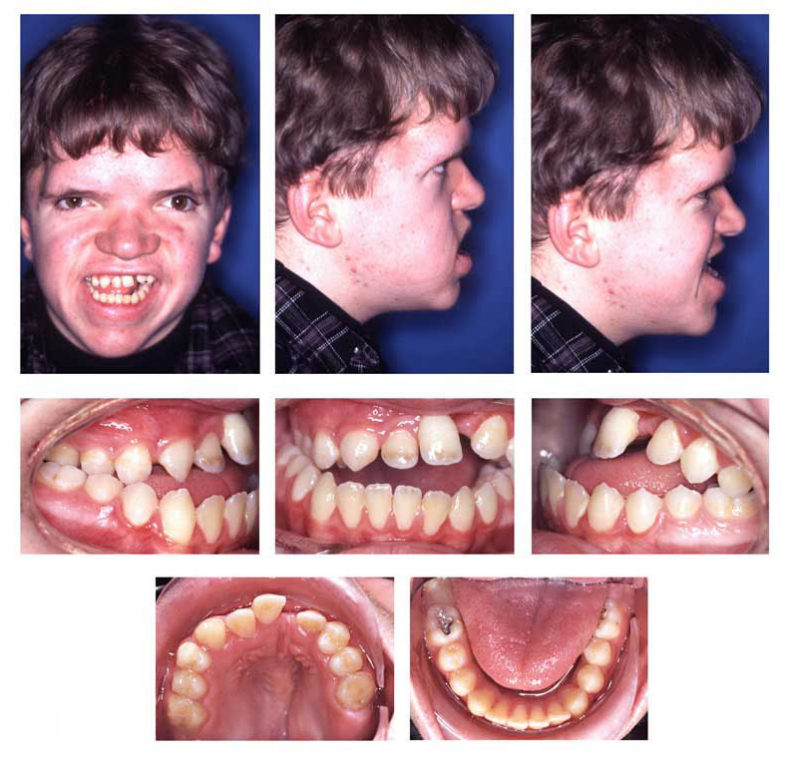
Age 15.1. One month before insertion of fixed appliance in maxilla. Extraorally: Hypertelorism, vertical excess of lower face impeding lip closure at rest, laterognathia, Class III. Intraorally: Class III, anterior open bite, unilateral crossbite, and midline deficiency.

**Figure 17:**
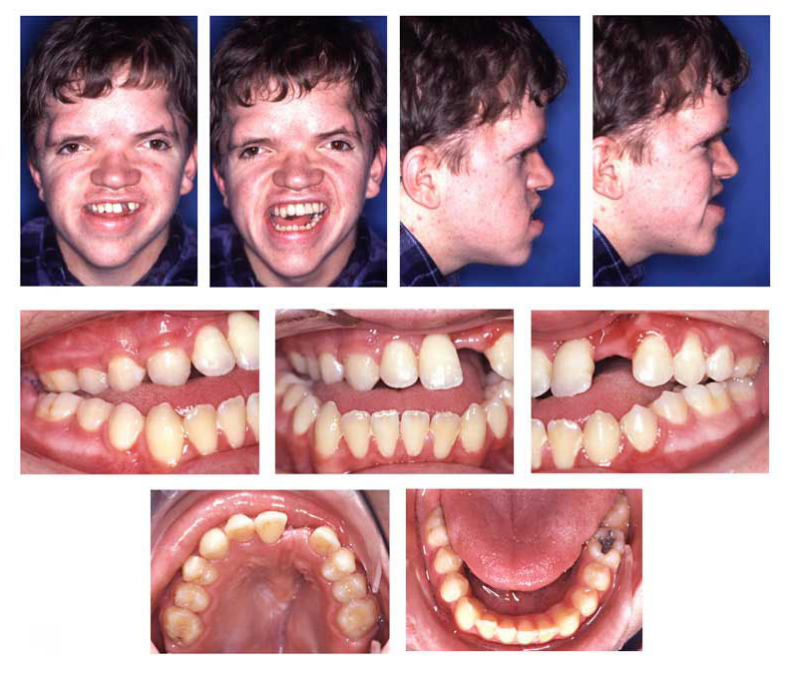
Age 16.4. Situation after 1.2 years in braces in order to parallelize 11 and 22 for later prosthodontic rehabilitation. Teeth 24 and 25 had not been bonded because of idiopathic root resorption (compare panographic x-ray, Fig. 16). Extraorally: With and without thermoplastic retainer for temporary replacement of 21. Intraorally: Teeth 11 and 22 were parallelized.

**Figure 18:**
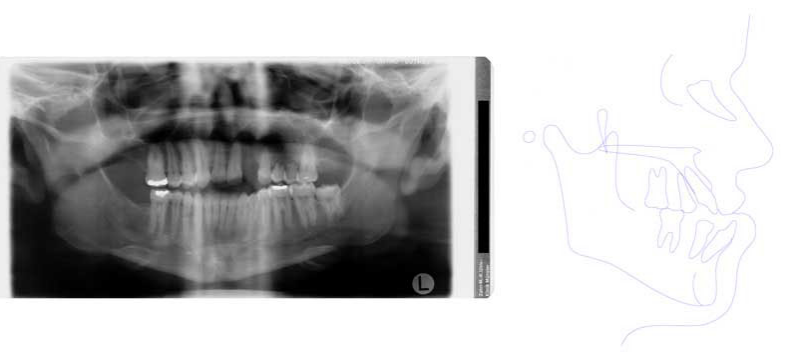
Left: age 18.2. Panographic x-ray 5 months before surgery. Right: age 18.6. Tracing of lateral x-ray before orthognathic surgery.

**Figure 19:**
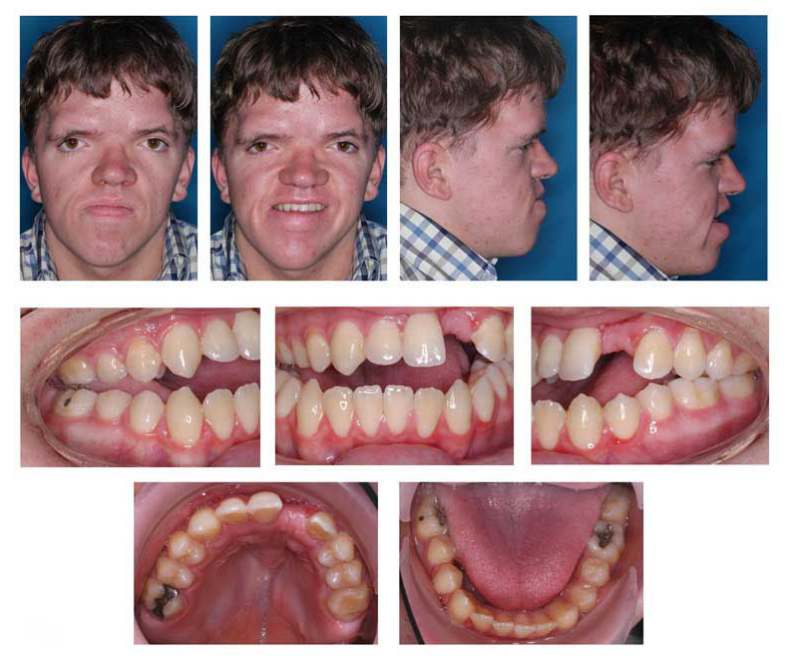
Age 18.6. Before orthognathic surgery. Extraorally: With thermoplastic appliance for temporary replacement of 21. Intraorally: Treatment result remained stable (compare Fig. 18).

**Figure 20:**
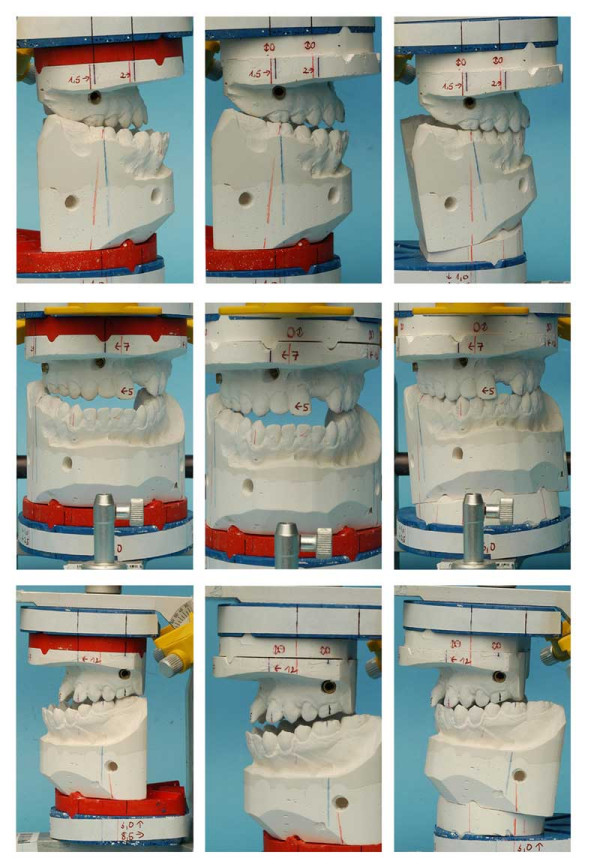
Age 18.6. Plaster surgery with KDMMS [20]: red plate in place = before plaster surgery; plaster splint in place = after plaster surgery. Right side of patient: before surgery, after plaster surgery in the maxilla (1.5–2 mm maxillary advancement), and after two-jaw plaster surgery (2.5 mm mandibular setback). Frontal view of patient: before surgery, after plaster surgery in the maxilla (5–7 mm maxillary rotation) (gap between maxillary cast and plaster splint at the left side of the patient is due to lack of antagonistic contact before plaster surgery of the mandible), after two-jaw plaster surgery (lift of the left side of the mandible). Left side of the patient: before surgery, after plaster surgery in the maxilla (12 mm maxillary advancement) (gap between maxillary cast and plaster splint at the left side of the patient is due to lack of antagonistic contact before plaster surgery of the mandible), after two-jaw plaster surgery (8.5 mm setback and 3 mm lift in the mandible).

**Figure 21:**
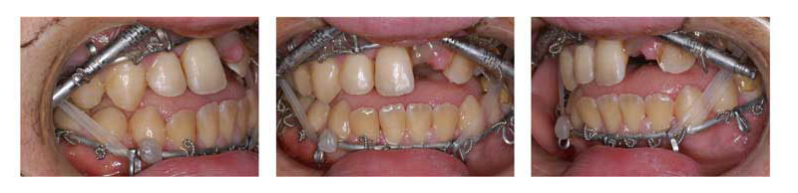
Age 18.6. After surgery, during distraction. Maxillary complex is "guided" by intermaxillary elastics during distraction phase. Bite planes have been mounted on 36 and 46, in order to overcome initial crossbite.

**Figure 22:**
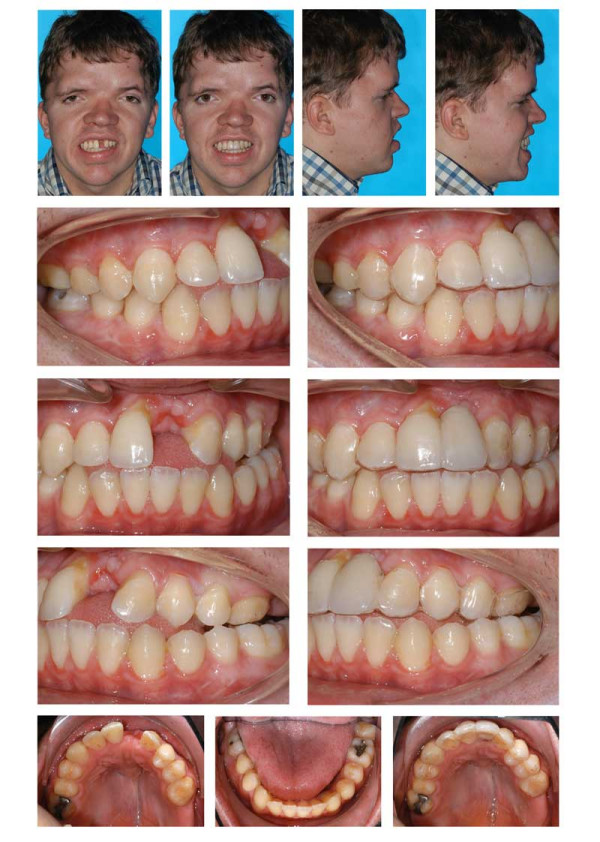
Age 19.7. With and without thermoplastic appliance for temporary replacement of 21. Note exact congruency of midline. Crossbite in region of teeth 24 and 25 corresponds to plaster surgery planning. Anteriorly, however, probably due to tongue thrust, a slight reopening tendency of the bite seems to have occurred. Logopedics (speech therapy) has been recommended for this reason.

**Figure 23:**
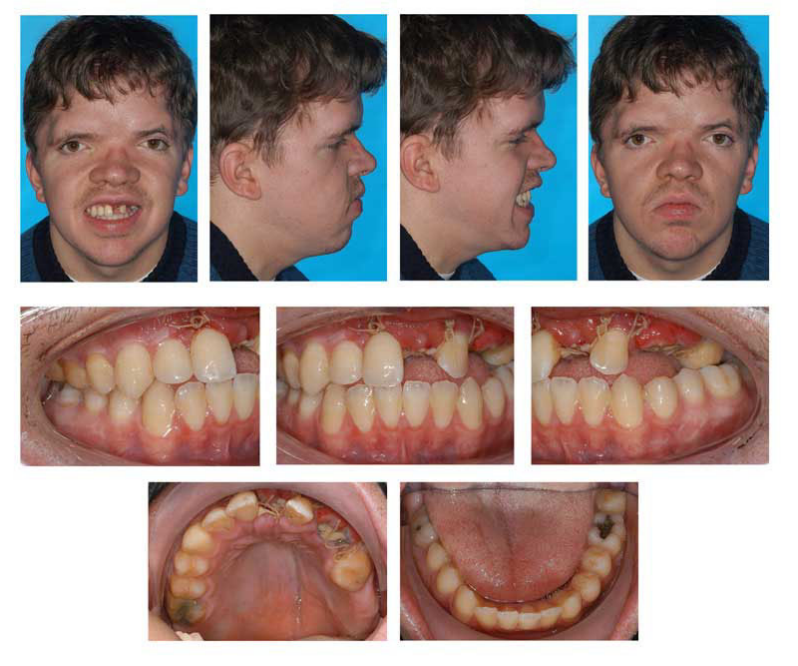
Age 20.2. After removal of 24 and 25 due to idiopathic root resorption and preparation for implants in region of 21, 24, and 25. Prosthodontic rehabilitation has not yet been completed.

## Discussion of exemplarily chosen cases

### Case 1

Although transposition of teeth 22 and 23 was accepted and torque of teeth 21 and 23 and occlusal contact of the right premolars could not be established, the patient was reported to have benefited considerably from the orthodontic treatment, both esthetically and functionally. Despite long distances for the teeth to move and 3 years of treatment, no root resorptions developed.

Thirty-six months' treatment was quite long as compared with nonsyndromic patients, nevertheless quite short considering the severity of the initial malocclusion. In case reports on Apert syndrome, Gugny [21,22] reported a treatment time of 33 months (with further treatment with fixed appliances advised); Rynearson [23] treated a patient with Crouzon syndrome for 58 months; and Matsumoto et al. [24] reported a treatment time of 10 years. In contrast to the present case, where an upper anterior tooth was transposed, Rynearson's patient came out with a more perfect result (having only one tooth in crossbite), which was achieved with a multisegmental LeFort I osteotomy (i.e., a surgical setup) and 61 % more orthodontic treatment time [23]. Gugny's result, after 33 months of orthodontic treatment, showed residual spaces, rotations, midline deficiency and crossbite [21,22]; nevertheless, the improvements of the initial malocclusion were great.

The need for extractions in patients with Apert syndrome is described in various articles [21-23,25]. The teeth selected for extraction, however were different from the present case, where unerupted canines were extracted: Gugny [21,22] and Gugny and Vi Fane [25] decided to extract the upper lateral incisors and premolars in cases, where the canines already had erupted; Rynearson [25] extracted one premolar and several molars, but in his patient with Crouzon syndrome, too, the canines already had erupted.

In 1991, Ferraro [7] pointed out that certain patients with Apert syndrome might benefit from genioplasty to provide an orthognathous appearance, which has been proved by the present case.

An alternative to the practised compensating orthodontic treatment with genioplasty would have been surgical correction of the open bite. This might have reduced the total treatment time, but would have increased the patient's surgical risks.

The hand deformity and the decrease in arm mobility made it difficult to floss–even brush the teeth [7] which might explain the difficulties in maintaining oral hygiene. Another reason might be inanition due to the extended treatment time.

### Case 2

Practising orthodontic treatment phased to orthognathic surgery implies the risk of relapse and thus the need for grinding prior to surgery in patients who do not wear their retainers properly, but phasing orthodontic treatment to orthoganthic surgery might have psychologic benefits.

Orthognathic surgery implies certain risks, but it is sometimes necessary to improve a patient's appearance and social adjustment [26].

Problems in maintaining oral hygiene were comparable with those of case 1, probably for the same reasons.

Also comparable with case 1, case 2 also involved problems in establishing correct torque. Additional problems with lack of rotation of teeth and decelerated tooth mobility in general made the orthodontic treatment of case 2 more difficult, and led to a more compromised result as compared with case 1.

Treatment of case 2, like that of case 1 and for the same reasons, required other than the "traditional" extraction decisions.

For a discussion of the suggested orthognathic surgery, see case 3.

### Case 3

Initial tooth positions of case 3 were better than in cases 1 and 2. Orthodontic correction of the crossbite on the left side was impossible because of lack of possible anchors (primary absence of 21 after need for extraction due to idiopathic root resorption and additional idiopathic resorption of 24 and 25, prohibiting inclusion of these teeth into the appliance).

We agree with other authors that in patients with Apert syndrome, midface advancement will almost always be required, and repeat cranial surgery is usually necessary [7,17]. Midfacial surgery is also best postponed as long as possible, but many children require surgery at around 4 to 6 years of age [17]. Early midface advancement has anatomic and psychologic benefits [25] as well as functional advantages such as reduction of sleep apnea [27,28]. However, the earlier midface surgery is performed, the more likely it is that further operations will be needed [17]. Meazzini et al. [9] described in a cephalometric study that, after LeFort III osteotomy at younger than 10 years, the stability of the maxillary segment was remarkable with neither detrimental nor beneficial effects on subsequent growth. The authors concluded that the standard LeFort III osteotomy in childhood is a stable and effective–but generally not definitive–procedure. We do agree with that, i.e. to our experience an early LeFort III osteotomy implies the advantage to avoid a (second) LeFort III osteotomy in adolescence, but does not avoid a second osteotomy in general.

The lack of relapse and forward growth of the maxilla after surgery at younger than 10 years as described by Meazzini et al. [9] has been confirmed in a study by Fearon [28]. This author speculates, that after halo distraction, most patients will likely avoid the traditional second LeFort III.

Technically difficult transpositions, involving craniosynostosis in particular, can be treated much more successfully by means of distraction osteogenesis because of bypassing the limitations of the covering soft tissues and the need for osseous transplants, resulting in the capacity for larger movements [27,29]. Distraction osteogenesis has some further advantages, such as fewer hazardous complications, less scarring, and fewer bone defects [30].

To solve the dilemma of either the forward mobilization of the upper part of the midface being insufficient when one focuses on the final occlusion, or the occlusion being unsatisfactory when forward mobilization of the upper midface is sufficient, Satoh et al. [31] has proposed a dual distraction osteogenesis. This involves separating the midface into two portions and conducting the movements separately in the upper and lower segments in different amounts and vectors by means of internal and external distraction devices. A known problem with distraction of the midface attributed to incomplete osteotomy and muscular influence is not a linear translatory advancement, but a complex three-dimensional displacement [32]. Thus, in contrast to a "traditional" distraction osteogenesis, the dual distraction osteogenisis proposed by Satoh at al. [31] only seems to exaggerate this problem.

If a mandibular setback is planned, special care must be taken to avoid significantly decreasing the airway volume, as this surgical procedure might trigger obstructive sleep apnea pre-existing in patients without Apert syndrome [33,34].

### General aspects and summary of all cases

Despite intrasyndromic similarities, the phenotypical expression in patients with Apert syndrome is highly variable [4,10]. This clinical variability [18] requires creativity, both in treatment planning and treatment progress. The care offered by a multidisciplinary team [5,7,17,22] is indispensable.

Tooth eruption and tooth movement of all patients was decelarated in general. Despite various orthodontic and surgical measures, some teeth moved insufficiently or not at all. Ferraro [7] has already pointed out that tooth eruption might be decelerated secondarily to the "mesenchymal disorder" of Apert syndrome. Periodontal fibers might suffer from a disturbance in their reaction potential.

We do agree with Ferraro [7], that orthopedic devices for class III malocclusions will not work in patients with Apert syndrome. The same applies to palatal expension, which requires to be surgically assisted.

## Conclusion

With the present article, *problems *with respect to orthodontic and surgical procedures and interdisciplinary approach in patients with Apert syndrome are described for the first time.

Orthodontic treatment of patients with Apert syndrome is possible, and under reasonnable conditions. The outcome of interdisciplinary treatment – which is indispensable – might not necessarily be either normal appearance or function but the achievement of the best appearance and highest level of function possible.

Patients need to be informed about (1) the different kinds of extractions that might be necessary, (2) the increased treatment time, and (3) a realistic expectation of results.

## Competing interests

The author(s) declare that they have no competing interests.

## Authors' contributions

AH performed the orthodontic treatment, conceived the necessity for publication, and drafted the manuscript. UJ performed the surgery. UM was responsible for the distraction protocol. UJ, UM, UE, and TS revised the manuscript critically for important intellectual content. All authors read and approved the final manuscript.
